# Theoretical Studies Aimed at Finding FLT3 Inhibitors and a Promising Compound and Molecular Pattern with Dual Aurora B/FLT3 Activity

**DOI:** 10.3390/molecules25071726

**Published:** 2020-04-09

**Authors:** Ítalo Antônio Fernandes, Déborah Braga Resende, Teodorico Castro Ramalho, Kamil Kuca, Elaine Fontes Ferreira da Cunha

**Affiliations:** 1 Department of Chemistry, Federal University of Lavras, P.O. Box 3037, Lavras-MG 37200-000, Brazil; italofernad@hotmail.com (I.A.F.); teo@ufla.br (T.C.R.); 2 Department of Veterinary Medicine, Federal University of Lavras, P.O. Box 3037, Lavras-MG 37200-000, Brazil; deborahbrr@gmail.com; 3 Faculty of Science, Department of Chemistry, University of Hradec Kralove, Rokitanskeho 62, 500 03 Hradec Kralove, Czech Republic

**Keywords:** indolin−2-one derivatives, dual Aurora B/FLT3 inhibitors, computational chemistry

## Abstract

FLT3 and dual Aurora B/FLT3 inhibitors have shown relevance in the search for promising new anticancer compounds, mainly for acute myeloid leukemia (AML). This study was designed to investigate the interactions between human FLT3 in the kinase domain with several indolin−2-one derivatives, structurally similar to Sunitinib. Molegro Virtual Docker (MVD) software was utilized in docking analyses. The predicted model of the training group, considering nineteen amino acid residues, performed in Chemoface, achieved an R^2^ of 0.82, suggesting that the binding conformations of the ligands with FLT3 are reasonable, and the data can be used to predict the interaction energy of other FLT3 inhibitors with similar molecular patterns. The MolDock Score for energy for compound 1 showed more stable interaction energy (–233.25 kcal mol^−1^) than the other inhibitors studied, while Sunitinib presented as one of the least stable (–160.94 kcal mol^−1^). Compounds IAF70, IAF72, IAF75, IAF80, IAF84, and IAF88 can be highlighted as promising derivatives for synthesis and biological evaluation against FLT3. Furthermore, IAF79 can be considered to be a promising dual Aurora B/FLT3 inhibitor, and its molecular pattern can be exploited synthetically to search for new indolin−2-one derivatives that may become drugs used in the treatment of cancers, including AML.

## 1. Introduction

Feline McDonough Sarcoma (FMS)-like tyrosine kinase 3 (FLT3, EC: 2.7.10.1) is a class III receptor protein tyrosine kinase, wherein five immunoglobulin-like domains are present in the extracellular region [1]. Under normal conditions, this enzyme is expressed in the membranes of precursor hematopoietic cells and is important for cellular differentiation, proliferation, and multiplication. In acute myeloid leukemia (AML), blasts express FLT3, and deregulation of its signaling pathway leads to exacerbated cellular proliferation, either by hyperstimulation or mutations, both of which contribute to AML onset [2,3].

The FLT3 gene is highly affected in this disease, with mutations being observed in about 40% of AML cases [2]. Justamembranar domain mutations lead to a FLT3/ITD (internal tandem duplication) mutant allele, which has been associated with a decrease in leukemia remission of patients undergoing chemotherapy with a low response to standard cytotoxic agents [4]. Evidence shows that mutations in a justamembranar region interfere with the self-inhibitory mechanism, leading to a conformational modification and constitutive activation of this enzyme [1]. Bavetsias and Moore [5,6] showed that dual Aurora/FLT3 inhibitors might be more effective than selective FLT3 inhibitors in cases of AML mutations.

**Figure 1:**
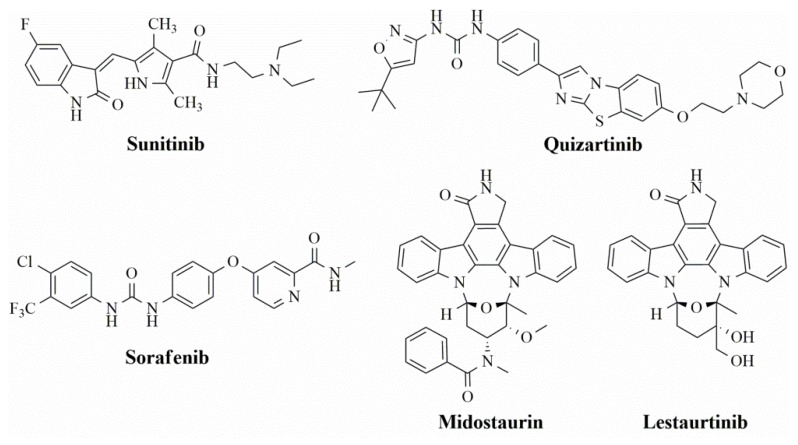
Chemical structures of Sunitinib, Quizartinib, Sorafenib, Midostaurin, and Lestaurtinib.

In the search for more efficient chemotherapic agents, FLT3/ITD inhibitors [7], such as Sunitinib (IC_50_ = 34.0 nM), Quizartinib (IC_50_ = 1.1 nM) [8], Midostaurin (IC_50_ = 9.3 nM), Lestaurtinib (IC_50_ = 8.6 nM), and Sorafenib (IC_50_ = 18.5 nM) [9] (Figure 1) have been discovered, and although presenting tolerability and toxicity problems, this has also inspired new studies in the search for new promising anticancer agents.

We reported on a study between indolin−2-one derivatives and human Aurora B kinase through molecular docking [10]. Now, in this work, we apply the same methodology looking forward to new potent FLT3 and Dual Aurora B/FLT3 inhibitors. Docking studies were performed in order to understand the binding mode of indolin−2-one derivatives, previously reported by Chern et al. [11], inside kinase domain FLT3. This was done to aid in the search for new Sunitinib analogues that may be more efficient and promising for the treatment of different cancer types, including AML.

## 2. Results

**Figure 2:**
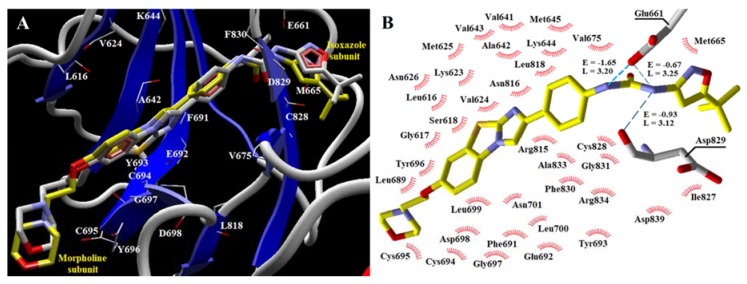
Quizartinib into the Feline McDonough Sarcoma (FMS)-like tyrosine kinase 3 (FLT3) interaction site. (**A**) The structure with yellow carbon atoms is the redocked Quizartinib. (**B**) Hydrogen bonds provide evidence for redocked Quizartinib and amino acid residues are located at a distance of 5Å.

Crystal coordinates of the FLT3 enzyme Quizartinib and the crystallographic water molecules were taken from the Protein Data Bank (PDB) [12]. At the interaction site, Quizartinib was redocked, and the conformation (Figure 2A) with the most appropriate spatial arrangement and most favorable energy parameters was selected (RMSD = 0.99Å) and applied as a reference compound for our docking studies. The analyzed site in docking studies was defined as a subset region around the center of the Quizartinib chemical structure in the human FLT3 enzyme, and the amino acid residues are shown in Figure 2B.

Compounds **1**–**41** and Sunitinib (Table 1) were docked into the FLT3 binding site using the MVD. The ligands and amino acid residues close to 5Å from the interaction site (Figure 2B) were considered flexible during the docking simulations. Thus, a candidate solution was encoded by an array of real-valued numbers representing the ligand position, conformation, and orientation as cartesian coordinates for ligand translation, whereas there are four variables specifying the ligand orientation and one angle for each flexible torsion angle in the ligand. MolDock Score energy values were obtained from each of the selected poses for all evaluated compounds, including Quizartinib and Sunitinib, and the values are expressed in kcal mol^−1^ (Table S1); energy values for pose–protein interactions and hydrogen bonds were also determined. The interaction modes of the ligand with the interaction site were determined to represent the lowest energy scored protein–ligand complex used during docking, and the conformers of each compound were mostly associated with conformations of redocked Quizartinib, which was the co-crystalized compound in then interaction site. Thus, it was used as the reference compound in our docking studies. The choice of the conformation of the ligand into active site was determined as the conformation with lowest energy scored of the protein–ligand complex used during docking and the position of each compound mostly associated with conformations of redocked Quizartinib (co-crystalized compound into interaction site). The total energy between each selected ligand conformation and the amino acid residues present at a distance of 5Å (those that exhibited some type of interaction: hydrogen bond, electrostatic or steric) for the training group was calculated and the results are shown in Table 2.

The model predicted was developed based on the training set (28 compound) and predictive potential was checked for the test set (12 compounds). The Kennard–Stone algorithm was employed to split the test dataset. Multivariate analyses such as partial least square were carried out for correlating descriptors (energy values) with observed activity (pIC_50_). The model was validated by validation techniques and the results are showed in Table 3.

**Figure 3:**
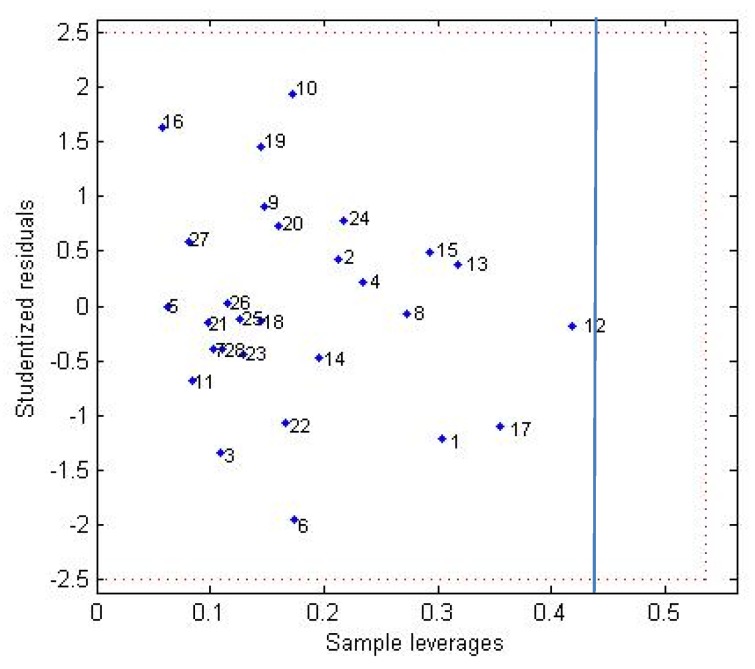
William’s plot.

The pIC_50_ values for the training and test set were computed and residual values were calculated and are reported in Table S2. The standard deviations (SD) of the residual values were 0.22 (training) and 0.31 (test). To establish outlier compounds, the residuals which were more than twice the SD of the residual of fit were observed. Our data showed that the training set has two compounds outlier and test set does not have outlier. In addition, the applicability domain of the model was assessed, using William’s plot, to identify possible atypical behavior (Figure 3). In this plot, the horizontal and vertical straight lines indicate the limits of normal values: the first for the outliers and second for influential compounds. A compound will be considered outside of the applicability domain when the leverage value is higher than the critical value of 3p/n (0.44), where p is the number of model variables plus 1 and n is the number of compounds used to develop the model. A value of 2.5 for standardized residual is commonly used as a cut-off value for accepting predictions. There are no compounds which lie outside of the applicability domain of the model.

## 3. Discussion

**Figure 4:**
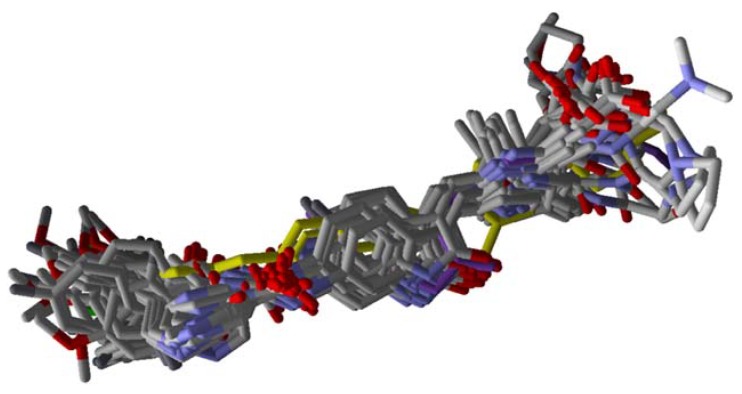
Overlapped conformations of FLT3 inhibitors. Quizartinib is shown in yellow, and Sunitinib is shown in purple.

**Figure 5:**
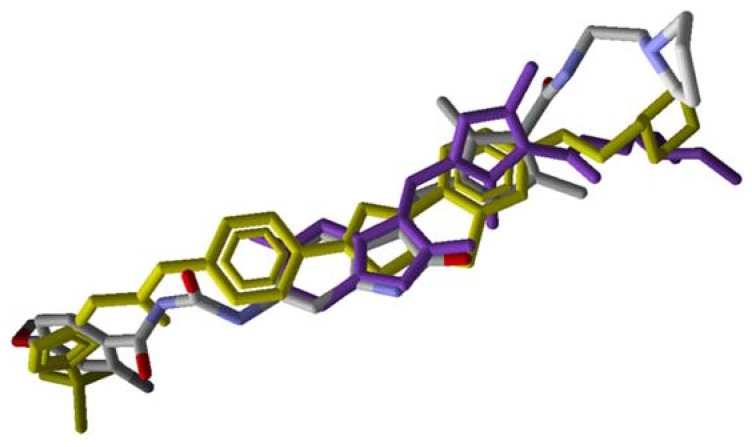
Overlapped conformations of compound **1**, Quizartinib (yellow), and Sunitinib (purple).

By analyzing the overlapping of compounds **1**–**41** and Sunitinib (Figure 4), the distribution pattern of the selected poses for these compounds can be verified, all following the alignment for the co-crystallized Quizartinib compound. Compound **1** showed a more stable interaction energy (−233.25 kcal mol^−1^) than the other inhibitors studied, while Sunitinib presented as one of the least stable (−160.94 kcal mol^−1^). The overlapping conformations for compound **1** and Sunitinib, compared to co-crystallized Quizartinib, can be visualized in Figure 5. Both exhibited good alignment with the reference compound, highlighting the fact that compound **1** fills, similarly, most of the positions occupied by Quizartinib.

Considering the results of Table 2, the great majority interact via hydrogen bonds, with Glu692 and Cys694 (–NH and carbonyl indolinone moieties, respectively). The following situations were observed: thirty-eight compounds with Cys694; thirty compounds plus Sunitinib with Glu692; and twenty-six compounds with both. Besides those, thirty-four compounds plus Sunitinib interacted with Cys694 or Leu616 residues through an NH–pyrrolic ring. Thus, Glu692 and Cys694 residues have an important contribution to the potency of these compounds, wherein indolin−2-one and pyrrolic ring groups are important subunits. In addition, hydrogen bond interactions were observed between the following:

i) Compounds **3**, **10**, **13**, **15**, **16**, **17**, **20, 21**, **24**, **25**, **28**, **30**, **31**, **34**, **36**, **38**, **40** and, Asp829 through pyridinone carbonyl;

ii) Other compounds also interacted with Asp829 residue through different groups: *N*-imidazolidinone (**37**, **41**); amidic carbonyl (**9**, **20**); NH between pyridinone and indolinone groups (**26**, **29**); and amidic carbonyl located next to the terminal benzene (**12**, **18**, **27**);

iii) Compounds **4**, **10**, **13**, **21**, **26**, **30**, **38** and Glu661 through NH-pyridinone;

iv) Compounds **2**, **6**, **8**, **15** and **18** with Leu616 and **4**, **11**, **27** and **35** with Tyr693 and Cys695 through the –COOH terminal group;

v) Other compounds also interacted with the Tyr693 residue through different groups: carbonyl group (**3** and Sunitinib) attached to the pyrrol ring; and *N*-pyrrolidine (compound **23**); N-morpholine (compound **32**);

vi) Compounds **9** and Cys828 interacted through amidic carbonyl between pyridinone and indolinone groups;

vii) Compounds **1**, **5**, **6**, **14**, **19**, **22**, **35** and **40** interacted with Asp829 and **12**, **18**, **23**, **27** and **33** interacted with Glu661 through the –NH group located between two acyclic carbonyls.

As observed by Zorn et al. [12], regarding redocked Quizartinib, hydrogen bond interactions were identified through NH-diaryl urea (Figure 2B)—two interactions with Glu661 (−1.65 kcal mol^−1^; −0.67 kcal mol^−1^) and another with Asp829 (−0.93 kcal mol^−1^) residues.

Analyzing the amino acid residues that presented the greatest energy ranges (Table 2) for compounds **1**–**41**, Quizartinib, and Sunitinib, Met665 and Glu661 can be highlighted. Sunitinib showed no interaction for both residues. For Met665, the most unfavorable energy values were observed for compounds **2** (70.68 kcal mol^−1^) and **7** (77.63 kcal mol^−1^), while for Glu661, unfavorable values were observed for compounds **18** (44.19 kcal mol^−1^) and **23** (32.16 kcal mol^−1^). These can be explained by the high structural proximity of one of the terminal moieties of these compounds, which generates high steric impairment levels as well as unfavorable electrostatic interactions. Observing the level of steric impairment, it can be verified that these are the two amino acid residues that lead the greatest steric impairment, of which Met665 is the largest, followed by Glu661. In contrast, by analyzing the most favorable values, compounds **30** (−4.64 kcal mol^−1^) and **41** (−4.84 kcal mol^−1^) for Met665 and compounds **12** (−25.33 kcal mol^−1^) and **33** (−21.55 kcal mol^−1^) for Glu661 were registered, orienting more appropriately and reflecting directly in interaction energy values, being considerably better for Glu661 due to having lower steric impairment and being a charged amino acid and thus achieving a greater number of favorable electrostatic interactions compared with Met665, an uncharged amino acid.

Another highlighted residue is Asp829, which showed a range of energy values between −8.60 kcal mol^−1^ (compound **2**) and −28.56 kcal mol^−1^ (compound **5**), but if Sunitinib was also considered, it would show the least favorable value (−2.84 kcal mol^−1^) among all compounds present in Table 1. This lower value for Sunitinib, but still negative and favorable, can be explained by the repulsion of charges between this negatively charged amino acid and the fluorine atom, despite the low steric impairment. For compounds **2** and **5**, it can be noticed that compound **2** showed a considerably more unfavorable orientation than compound **5**, which achieved high steric impairment and hydrogen bond absence.

For Phe691, the range of values varied from −20.81 kcal mol^−1^ (compound **20**) to +5.56 kcal mol^−1^ (compound **31**), which can be explained by the better overlap of the pyridinone subunit, present in both compounds, in compound **20**, with the benzenic subunit of this aromatic amino acid, thereby generating a more favorable energy value for compound 22 in contrast with compound **31**, which has been shown to be sterically hindered by this amino acid due to torsion in the pyridinone subunit.

The model used for the prediction of activities of FLT3 inhibitors depends on statistical significance and predictive ability. Table 3 showed the results generated, an R2 value of 0.81 (RMSEc = 0.29) was achieved from calibration. The R2 values greater than 0.8 indicates that the model is correlated and may be considered to represent the training set in the same manner. A q2 of 0.60 (RMSEcv = 0.40) reveals that the model can be a useful tool for predicting affinities of new compounds based on these structures. In respect to y-randomization, the model has R^2^ higher than R^2^rand (0.22) and R^2^p (0.62) higher than 0.5, thereby assuring the inexistence of chance correlation. Therefore, it can be concluded that the developed model has good performance, with suitable goodness of fit, robustness and predictivity.

The equation coefficients from the regression analysis may provide useful information: the terms with positive coefficient signs decrease the predicted potency of a compound. The same concept can be applied to terms with negative coefficient signs that increase the predicted potency. In both cases, the predicted potency values are directly proportional to the magnitude of each coefficient term of this equation. The predicted pIC_50_ values for training were computed and are shown in Table S2. The standard deviation (SD) of the residual values was 0.26. To establish outlier compounds, we observed which residuals were more than twice the SD of the residual of the fit. Analyses of the data showed one outlier, compound 1, but due to its better activity compared with the other compounds and its use as a molecular pattern for searching for new promising analogues, it was considered for the predictive model.

Lys644 contributes to a decrease in the interaction potency of compounds and presents the highest positive regression coefficient (0.132). This positively charged amino acid is located near the terminal subunit, wherein the majority of compounds **1**–**41** have a substituted benzene or pyridinone. In the case of Quizartinib, it is next to the isoxazole group, and Sunitinib has no interaction. It is verified that repulsive electrostatic interactions occur with positively charged atoms in the molecules, as does steric impairment. Its distance is higher when compared with other residues in the interaction site and can be observed in Figure 2A, thus generating a lower influence on interactions for the evaluated compounds. Residues that also showed positive regression coefficient values were Phe691 (0.030), Glu661 (0.025), and Met665 (0.017), which all showed interaction energy values in a wide range (Table 2).

Val624 was shown to contribute to the increase in the interaction potency of the compounds and presented the most negative regression coefficient (–0.204). This amino acid is close to the indolin−2-one and pyrrol groups (compounds **1**–**45** and Sunitinib), except in compound **22** which has a furane, and the central phenyl-benzoimidazothiazole subunit for the Quizartinib compound (Figure 2A). All of these compounds exhibited favorable hydrophobic interactions with this uncharged, non-polar amino acid residue and there was an absence of highly unfavorable steric or electrostatic interactions. Two other residues that can be pointed to with negative regression coefficient values were Glu692 (–0.176) and Leu818 (–0.146).

By structurally analyzing pyridinone, imidazolidinone, and acyclic diamide in compounds **1**–**45**, it was verified that pyridinone generates a greater steric impairment between the three groups, therefore it is a group that can be structurally optimized. However, an acyclic subunit was maintained, as in the case of the diaryl urea group in Quizartinib. Furthermore, imidazolidinone was not as sterically unfavorable as pyridinone, but the acyclic diamide or diaryl urea still seem to be the most favorable, possibly due to their greater conformational freedom.

When compound **1** and Quizartinib are compared using the interaction energy values (kcal mol^−1^) of each amino acid residue, the widest energy variations were shown in Asp829, Cys694, Cys695, Leu616, Phe691, Phe830, Tyr693, Tyr696, Val624, and Val675. All nineteen important amino acid residues appeared in both compound **1** and Quizartinib. When comparing compound 1 and Sunitinib, since compounds **1**–**41** can all be considered structural optimizations of this one, the widest energy variations were shown in Asp829, Cys695, Cys828, Glu661, Leu616, Lys644, Met665, Phe691, Tyr696, and Val675 residues, of which amino acid residues Glu661, Lys644, and Met665 showed no interactions.

Through observations from docking studies, the same one hundred new indolinonic derivatives proposed by Fernandes et al. [10] were evaluated by applying our current predictive model, as well as carrying out observations of related parameters (MolDock scores, pose–protein interactions, and hydrogen bond energy values). Among the one hundred indolinonic derivatives, satisfactory poses were obtained in eighty-one derivatives, and all of them showed interactions in the nineteen defined amino acid residues. Six compounds (IAF70, IAF72, IAF75, IAF80, IAF84, and IAF88) can be highlighted and considered to be more promising for future synthesis and biological evaluations against the human FLT3 enzyme. These six compounds have a benzyle moiety attached to an *sp*2 carbon atom located between indolin−2-one and a pyrrolic ring, suggesting the filling of an important verified vacancy, leading to additional interactions that would considerably contribute to the inhibitory activity of these compounds. It is also noted that the presence of a benzyle moiety would be more important than the presence of a phenyl moiety for these compounds, as verified for the Aurora B target [10]. Furthermore, the IAF75 compound presented the most satisfactory set of parameters among the six most promising compounds, as follows: predictive activity (pIC_50_ = 10.24), MolDock Score (−257.27 kcal mol^−1^), Lig-Prot. (−254.38 kcal mol^−1^), and hydrogen bond (−13.95 kcal mol^−1^). This is shown in Table 4. Seven hydrogen bond interactions were observed for IAF75: Asn701, Asp698, Asp829, Cys694, Glu692, Gly697, and Tyr693 residues. IAF84 can also be highlighted, as it presented the lowest MolDock Score value (−261.42 kcal mol^−1^) and pose–protein interaction energy (−258.18 kcal mol^−1^), in addition to a considerable hydrogen bond energy (−11.16 kcal mol^−1^) for seven amino acid residues—Asn701, Asp698, Asp829, Cys694, Glu692, Leu616, and Tyr693—although it did not show the highest predicted activity.

**Figure 6:**
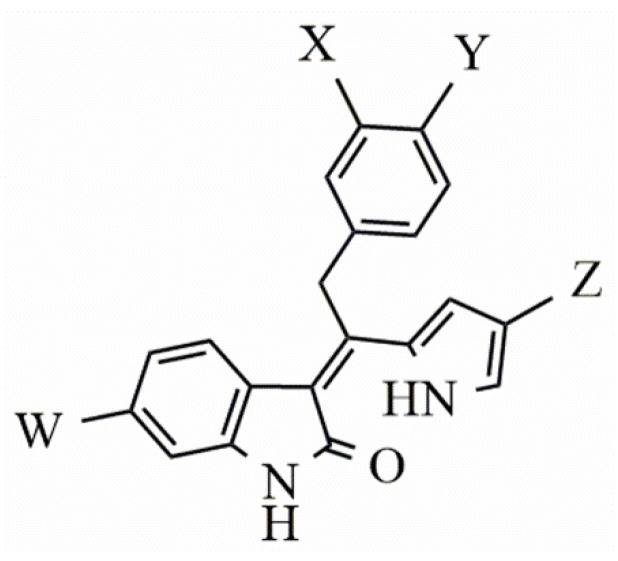
Promising indolin−2-one molecular pattern for dual Aurora B/FLT3 activity.

Considering studies conducted by our research group in the search for new promising inhibitors of Aurora B kinase [10] and the current results, the **IAF79** compound (Table 5) can be considered to be a promising dual Aurora B/FLT3 inhibitor aimed at the treatment of different cancer types, including myeloid leukemia, as it presents considerable predicted activity and energy values for both targets studied. As shown by its chemical structure, this compound, an analogous structural variation of compound **1** (Table 1), has an insertion of 4-ethylphenol or the *p*-cresol moiety attached in the *sp2* carbon atom located between the indolin−2-one and pyrrolic groups, leading to additional and favorable interactions in both Aurora B and FLT3 therapeutic targets; thus, it fills an important vacancy in both enzymes. Another structural difference observed was the absence of two methyl groups attached to the pyrrole ring that generate steric hindrance to the 4-ethylphenol group inserted, which could limit its conformational freedom during interactions with the targets in question. Thus, its molecular pattern (shown in Figure 6) can be exploited synthetically at different positions, especially at X, Y, W, and Z, and may aid in the search for new indolin−2-one derivatives with dual Aurora B/FLT3 activity that may become drugs used in the treatment of different types of cancer in the future.

## 4. Materials and Methods

### 4.1. Data Set

Enzyme crystal coordinates (organism: *Homo sapiens*) were downloaded from the Protein Data Bank (PDB code: 4XUF) [12]. Compounds **1**–**45** [11], Quizartinib, and Sunitinib FLT3 inhibitor (Table 1) were docked into the FLT3 kinase domain using the Molegro Virtual Docker (MVD) [13,14,15]. MVD is a program for predicting the most likely conformation of how a ligand will bind to a macromolecule. The MolDock Scoring Function (MolDock Score) employed by the MVD program is regulated by a new hybrid search algorithm, called guided differential evolution. This algorithm combines the differential evolution optimization technique with a cavity prediction algorithm during the searching procedure, which allows fast and accurate recognition of potential binding modes—the poses. It is derived from the Piecewise Linear Potential (PLP), a simplified potential whose parameters are fit with protein–ligand structures and binding data scoring functions [15] and further extended in the GEMDOCK program (Generic Evolutionary Method for molecular DOCK) with a new hydrogen bond term and new charge schemes. The docking scoring function, E_score_, is defined by the following energy terms:

(1) $$E_{\mathrm{score}\;}={\;E}_{\mathrm{inter}}+{\;E}_{\mathrm{intra}}$$

where the ligand–protein interaction energy, E_inter_, is given by

(2) $$E_{\mathrm{inter}}=\begin{array}{c} \sum \\ i\in \mathrm{ligand} \end{array}\begin{array}{c} \sum \\ j\in \mathrm{protein} \end{array}\left[E_{\mathrm{PLP}}\left(r_{\mathrm{ij}}\right)+332.0\;\frac{q_{i}{\;q}_{j}}{4r_{\mathrm{ij}}^{2}}\right]$$

The EPLP term is a “piecewise linear potential” that uses two different set of parameters: one set for approximating the steric term between atoms (van der Waals) and another stronger potential for hydrogen bonds. The second term describes the electrostatic interactions between charged atoms. It is a Coulomb potential with a distance-dependent dielectric constant, D(r) = 4r. The numerical value of 332.0 fixes the units of the electrostatic energy to kilocalories per mole (Molegro ApS).

The E_intra_ terms represent the internal energy value of the ligand:

(3) $$E_{\mathrm{intra}}=\begin{array}{c} \sum \\ i\in \mathrm{ligand} \end{array}\begin{array}{c} \sum \\ j\in \mathrm{ligand} \end{array}{\;E}_{\mathrm{PLP}}\left(r_{\mathrm{ij}}\right)+\begin{array}{c} \sum \\ \mathrm{flexiblebonds} \end{array}\;A\;\left[1-\cos\left(m.\theta \;-{\;\theta }_{0}\right)\right]+{\;E}_{\mathrm{clash}}$$

The first term, double summation, is the energy between all atom pairs in the ligand, excluding atom pairs, which are connected by two bonds. The second term is a torsional energy term, where θ is the torsional angle of the bond. The average torsional energy bond contribution is used if several torsions can be determined. The last term, E_clash_, assigns a penalty of 1000 if the distance between two heavy atoms is less than 2.0 Å, punishing infeasible ligand conformations (Molegro ApS). The docking search algorithm used in MVD is based on interaction optimization techniques conducted by Darwinian Evolution Theory (evolutionary algorithms, EA). A population of individuals is exposed to competitive selection that weeds out poor solutions. Recombination and mutation are used to create new solutions [15,16,17,18,19,20,21,22].

### 4.2. Predicted Model

The prediction model was constructed considering the interaction energies from docking for the nineteen most relevant amino acid residues located at a distance of 5 Å from the evaluated interaction site and the biological activity against FLT3 according to Chern et al. [11]. Subsequently, Chemoface software version 1.61 was used toward the statistical analysis:

i) Coefficient of determination (R^2^): is the proportion of the variance in the dependent variable that is predictable from the independent variable.
ii) Leave-one-out cross-validation (LOOcv) correlation coefficient (q^2^): estimating the performance of a predictive model.
iii) Y-Randomization (R^2^rand): consists of the random exchange of the independent variable values. Thus, the R^2^rand value must be less than the correlation coefficient of the non-randomized models.
iv) R^2^p: penalizes the model for the difference between R^2^ of randomized models and the R^2^ of the non-randomized model:
  (4) $$R_{P}^{2}=R^{2}\times \sqrt{R^{2}-R_{\mathrm{rand}}^{2}}$$
v) Correlation coefficient of external validation set (R^2^_pred_): reflects the degree of correlation between the observed (Y_Exp(test)_) and predicted (Y_Pred(test)_) activity data of the test set:
  (5) $$R_{\mathrm{Pred}\;}^{2}=1-\frac{\sum_{1}^{n\;}\;{\left(Y_{\mathrm{Exp}\left(\mathrm{test}\right)}-Y_{\mathrm{Pred}\left(\mathrm{test}\right)}\right)}^{2}}{\sum_{1}^{n}\;{\left(Y_{\mathrm{Exp}\left(\mathrm{test}\right)}-{\;\overset{\text{¯}}{Y}}_{\mathrm{training}}\right)}^{2}}$$
  where ${\overset{\text{¯}}{Y}}_{\mathrm{training}}$ is average value for the dependent variable for the training set.
vi) Modified R^2^ (R^2^_m(test)_): equation determining the proximity between the observed and predicted values with the zero axis intersection:
  (6) $$R_{m}^{2}=r^{2}(1-\left|\sqrt{R^{2}-{\;R}_{0}^{2}}\right|)$$

### 4.3. Searching for More Potent Indolin−2-one FLT3 Inhibitors

In our previous study, we performed a search for new promising inhibitors of human Aurora B kinase [10]. Using the same methodology and the same one hundred compounds, we looked for promising inhibitors for human FLT3, considering the orientations of compounds inside the interaction site compared with the co-crystallized compound Quizartinib. These derivatives were evaluated in silico, being submitted to docking studies in MVD, and, subsequently, a pre-established predictive model was applied. The selected compounds for the predictive model were all those that presented adequate poses, comparing to redocked Quizartinib with a satisfactory RMSD value. Thus, by getting the Quizartinib redocked conformation in the interaction site under study, the compounds were selected, considering satisfactory conformations and energy parameters. Interactions in most relevant amino acid residues occurring at a distance of 5 Å from the site of enzyme interaction were considered. By searching for potential promising inhibitors for the human FLT3 enzyme, besides the predictive model, the following energetic docking parameters were considered: MolDock Scores, protein–ligand interactions, and hydrogen bonds.

## 5. Conclusions

We carried out molecular docking studies in order to understand the interactions of a variety of indolin−2-one derivatives with the human FLT3 enzyme. The different substituent groups were exploited at positions 3 and 6 of the indolinone ring and are shown in Table 1. Docked structures were evaluated based on their binding energy and electrostatic and hydrogen bond interactions. Our molecular docking results, combined with experimental data for human FLT3 enzyme inhibition, suggest the presence of important empty space around the indolin−2-one moiety. Thus, more favorable substituents attached at the 3-position of the pyrrolic ring and in the *sp2* carbon atom located between the indolin−2-one and pyrrolic ring groups may increase the affinity for the human FLT3 enzyme, which was verified to be of a similar form to the Aurora B kinase by Fernandes et al. [10] using the same benzyle moiety. For new **IAF1**–**IAF100** compounds, six of them could be detached (**IAF70**, **IAF72**, **IAF75**, **IAF80**, **IAF84,** and **IAF88**). These all have a benzyle group attached in the *sp2* carbon atom located between the indolin−2-one and pyrrolic ring groups, and **IAF75** presented the most satisfactory set of parameters, while **IAF84** presented the lowest MolDock score and pose–protein interaction values. Moreover, considerable hydrogen bond energy was added to seven different amino acid residues.

The present study, together with studies by Fernandes et al. [10] on this class of indolinone compounds, seem to suggest that future obtainment of a candidate for an innovative drug for the treatment of different cancer types, including myeloid leukemia, is possible, since some compounds, particularly **IAF79**, have been shown to exhibit possible promising dual activity against Aurora B/FLT3 and have promising molecular patterns that can be further investigated synthetically in the search for new anticancer drug candidates to build on studies that have already been carried out.

## Figures and Tables

**Table 1:** Chemical structures, IC_50_ and pIC_50_ values of **1**–**41** [11], Quizartinib and Sunitinib FLT3 inhibitor compounds. Test set compounds are marked with an asterisk.

Compound	Chemical Structure	IC_50_ (nM) FLT3	pIC_50_ FLT3
**1**	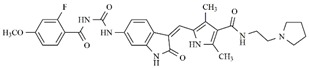	0.5	9.301
**2**	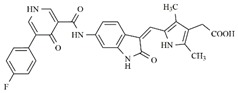	1.3	8.886
**3**	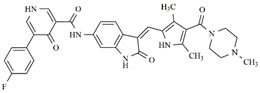	1.4	8.854
4*	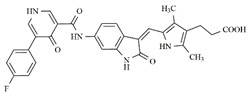	1.6	8.796
5*	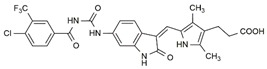	2.4	8.620
6*	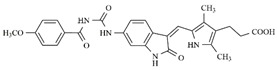	2.7	8.569
**7**	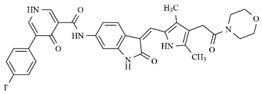	2.7	8.569
**8**	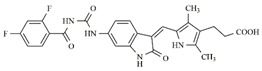	2.7	8.569
**9**	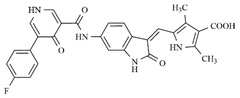	2.9	8.538
**10**	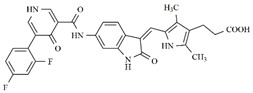	2.9	8.538
**11**	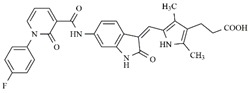	3.5	8.456
**12**	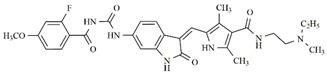	4.4	8.357
13*	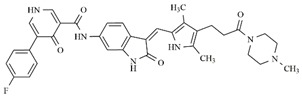	4.8	8.319
14*	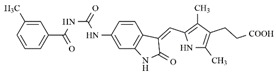	6.1	8.215
**15**	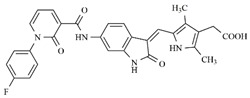	7.3	8.137
16*	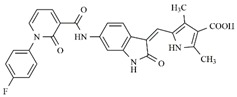	8.1	8.092
**17**	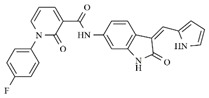	8.6	8.066
**18**	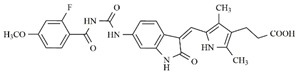	10.0	8.000
19*	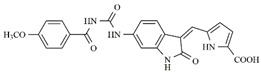	10.0	8.000
**20**	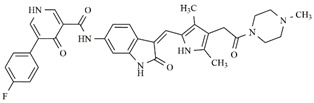	10.3	7.987
**21**	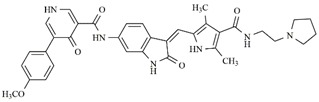	11.6	7.936
22*	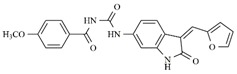	15.4	7.812
**23**	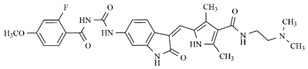	16.2	7.790
**24**	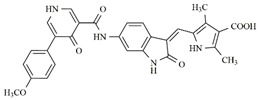	24.6	7.609
**25**	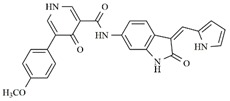	24.9	7.604
**26**	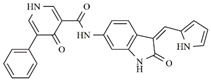	27.7	7.558
**27**	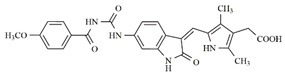	31.7	7.499
**28**	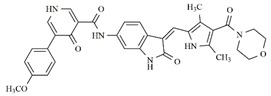	34.9	7.457
**29**	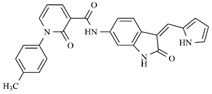	37.5	7.426
**30**	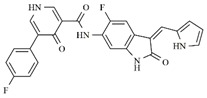	37.9	7.421
31*	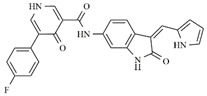	38.1	7.419
**32**	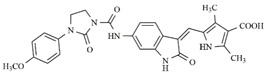	39.4	7.404
**33**	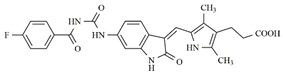	45.7	7.340
34*	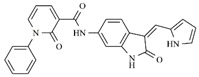	48.4	7.315
**35**	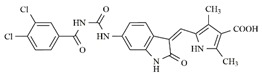	54.2	7.266
**36**	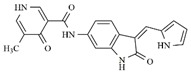	64.2	7.192
**37**	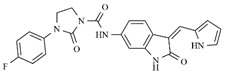	74.1	7.130
38*	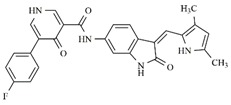	86.6	7.062
39*	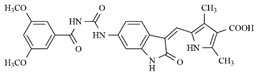	115.3	6.938
**40**	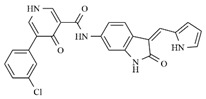	148.4	6.828
**Quizartinib**	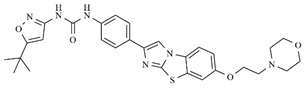	4.2 ^a1^ 1.1 ^a2^	8.377 8.959
**Sunitinib**	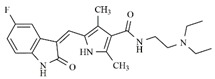	9.9 ^a1^ 34.0 ^a2^	8.004 7.468

^a1^: FLT3/Wild [8]; ^a2^: FLT3/ITD [8]. * Test set compounds.

**Table 2:** Interaction energy values (kcal mol^−1^) between amino acid residues and ligand for training and test set.

Cpd/aa	Ala 642	Asp 698	Asp 829	Cys 694	Cys 695	Cys 828	Glu 661	Glu 692	Gly 697	Leu 616	Leu 818	Lys 644	Met 665	Phe 691	Phe 830	Tyr 693	Tyr 696	Val 624	Val 675
**1**	−1.90	−1.91	−28.29	−12.11	−3.75	−18.82	−10.10	−4.10	−10.57	−23.07	−11.32	−1.91	4.22	−14.37	−8.25	−14.65	−4.19	−3.53	−10.21
**2**	−3.47	−2.52	−8.60	−11.61	−3.78	−8.46	11.60	−4.30	−9.89	−23.55	−12.07	−9.62	70.68	−12.02	−8.44	−12.14	−3.83	−5.74	−7.10
**3**	−3.18	−2.03	−14.63	−10.25	−8.59	−8.33	−21.14	−1.75	−10.32	−21.16	−13.13	−1.05	27.99	−12.19	−9.04	−13.02	−10.30	−4.48	−4.60
**4**	−2.06	−1.81	−12.19	−11.28	−8.05	−9.41	−15.47	−1.70	−9.61	−16.22	−14.06	−3.23	1.95	−4.00	−12.28	−13.12	−6.78	−5.76	−8.14
**5**	−3.93	−4.79	−28.56	−10.96	−3.05	−24.34	−7.84	−4.92	−11.72	−14.22	−12.61	−2.69	2.79	−14.80	−9.70	−10.11	−5.02	−5.04	−8.56
**6**	−3.72	−3.25	−19.77	−12.07	−2.87	−21.27	−4.86	−4.94	−9.70	−24.01	−11.81	−2.57	7.50	−12.93	−8.96	−10.96	−2.66	−5.69	−10.26
**7**	−3.58	−5.82	−9.43	−12.55	−3.54	−10.52	0.52	−2.20	−10.90	−17.27	−11.75	−7.18	77.63	−8.56	−7.08	−10.75	−6.27	−5.58	−4.75
**8**	−4.03	−5.13	−15.80	−12.87	−2.02	−23.44	−2.34	−5.47	−8.53	−20.14	−12.68	−1.61	9.50	−14.83	−8.84	−9.66	−2.13	−5.05	−7.23
**9**	−5.14	−2.17	−11.28	−8.87	−3.82	−6.94	3.47	−3.14	−10.13	−18.14	−9.58	−6.53	65.61	−12.54	−7.25	−11.71	−4.78	−8.17	−5.69
**10**	−2.30	−1.62	−12.27	−11.99	−3.68	−12.31	−9.30	−5.20	−9.17	−21.41	−11.99	−4.35	38.66	−3.53	−7.94	−12.27	−3.17	−3.58	−8.62
**11**	−2.39	−1.70	−13.05	−12.58	−9.24	−10.14	−16.87	−2.00	−9.45	−11.96	−14.02	−2.97	3.85	−2.70	−11.56	−15.15	−7.67	−5.44	−8.52
**12**	−3.22	−9.77	−17.90	−10.84	−4.04	−11.92	−25.33	−3.83	−15.35	−15.56	−12.73	−4.41	15.24	−14.71	−9.85	−10.81	−7.82	−5.72	−6.56
**13**	−1.79	−1.59	−13.55	−10.83	−6.14	−11.91	−8.55	−2.41	−9.81	−15.30	−13.45	−5.53	−4.10	−2.98	−10.12	−15.00	−6.59	−5.87	−7.99
**14**	−2.15	−1.80	−25.45	−12.12	−3.52	−19.77	−8.72	−5.03	−9.32	−21.43	−11.89	−1.71	3.03	−16.56	−8.81	−12.82	−3.25	−3.42	−7.48
**15**	−2.66	−2.27	−10.52	−12.48	−4.21	−10.40	−1.77	−3.69	−10.22	−24.67	−12.05	−6.20	54.98	−1.02	−7.62	−11.22	−4.02	−3.64	−8.03
**16**	−2.23	−1.92	−13.13	−13.07	−3.91	−10.60	−1.06	−4.51	−10.82	−17.85	−11.47	−5.12	39.50	−7.34	−7.64	−11.77	−4.50	−3.50	−9.17
**17**	−3.33	-0.91	−15.54	−14.26	−2.48	−12.12	8.11	−4.44	−6.15	−12.24	−11.87	−7.22	34.52	−3.82	−6.98	−12.19	−1.25	−5.27	−7.94
**18**	−3.48	−5.04	−20.27	−11.13	−2.16	−12.32	44.19	−1.95	−9.89	−22.54	−10.40	−2.13	−2.50	−18.51	−11.80	−7.48	−2.21	−4.43	−7.12
**19**	−2.15	−1.32	−25.70	−14.60	−2.07	−18.54	−9.08	−6.09	−6.94	−17.34	−11.95	−2.47	2.22	−16.83	−7.05	−12.42	−1.64	−4.73	−9.06
**20**	−2.94	−1.97	−12.01	−12.66	−6.93	−11.42	−17.81	−4.69	−14.44	−16.58	−10.88	-0.31	33.24	−20.81	−8.59	−11.82	−14.06	−2.89	5.41
**21**	−1.80	−1.48	−10.81	−11.82	−7.05	−11.32	−12.80	−1.94	−6.00	−19.99	−13.32	−4.11	-0.97	−3.64	−11.52	−6.08	−3.90	−6.15	−7.82
**22**	−1.70	−1.31	−12.20	−14.35	−1.81	−23.16	−2.52	−6.30	−4.04	−10.48	−11.75	−2.02	8.27	−13.68	−6.37	−10.84	−1.64	−5.55	−6.99
**23**	−2.59	−1.66	−18.36	−11.64	−3.54	−12.63	32.16	−3.09	−9.04	−13.48	−12.99	−7.69	−2.75	−19.03	−8.37	−14.20	−3.84	−3.67	−8.31
**24**	−4.49	−2.12	−13.80	−9.24	−3.64	−11.07	7.10	−3.21	−10.41	−18.62	−11.74	−9.56	27.49	−10.24	−8.61	−11.81	−4.27	−6.65	−7.45
**25**	−4.44	−1.16	−23.81	−11.81	−3.31	−11.65	2.30	−2.16	−4.03	−10.48	−12.26	−8.10	−1.23	11.81	−8.98	−11.76	−2.85	−6.58	−8.21
**26**	−3.46	−1.01	−17.06	−12.26	−2.70	−15.76	5.74	−1.79	−5.98	−10.60	−12.48	−3.40	50.76	−13.89	−7.20	−10.54	−2.08	−6.03	−1.60
**27**	−3.04	−1.25	−21.42	−10.60	−3.94	−11.96	−21.37	−4.68	−8.62	−15.67	−11.82	−4.61	−1.05	−14.40	−9.63	−16.59	−3.29	−6.16	−7.27
**28**	−1.88	−1.12	−16.49	−8.57	−6.61	−10.78	−7.95	-0.93	−7.19	−21.41	−10.72	−6.68	4.37	0.54	−9.77	−12.12	−4.89	−8.58	−7.27
**29**	−2.58	-0.85	−19.75	−12.76	−3.21	−10.32	12.68	−1.89	−6.22	−13.12	−11.82	−4.08	29.51	−12.09	−6.24	−12.57	−2.05	−6.41	−4.56
**30**	−1.89	-0.79	−14.09	−13.14	−2.10	−11.77	−6.60	−5.49	−5.51	−5.58	−12.82	−4.48	−4.64	−8.71	−11.04	−11.76	−1.12	−4.29	−7.49
**31**	−3.00	-0.67	−20.12	−13.76	−2.70	−11.78	−4.00	−4.92	−5.82	−10.84	−11.69	−6.47	−2.56	5.56	−7.77	−12.88	−1.14	−5.24	−8.90
**32**	−3.59	−2.33	−17.04	−10.53	−4.09	−15.48	−15.18	−1.60	−10.97	−18.08	−11.57	−1.90	6.44	−16.41	−8.20	−9.10	−5.18	−7.44	4.76
**33**	−2.96	−2.01	−14.00	−11.59	−3.68	−12.27	−21.55	−3.87	−10.00	−19.56	−12.71	−3.81	21.37	−16.47	−8.23	−12.49	−4.03	−4.28	−5.11
**34**	−2.04	−1.24	−14.85	−14.31	−2.46	−12.11	6.84	−5.96	−5.13	−8.97	−12.32	−5.81	27.07	−12.89	−6.44	−11.51	−1.79	−4.35	−8.87
**35**	−2.42	−1.55	−25.07	−11.15	−9.38	−18.03	−8.01	−2.73	-0.36	−13.27	−12.19	−1.68	−2.38	−15.94	−7.62	−16.61	−6.66	−4.76	−4.90
**36**	−2.07	−1.14	−14.32	−14.53	−1.87	−11.28	10.54	−5.39	−5.15	−8.90	−12.44	−7.02	2.20	−10.78	−7.14	−10.95	−1.30	−4.78	−8.37
**37**	−2.23	−1.22	−17.23	−14.97	−1.41	−20.78	−5.56	−6.80	−5.08	−10.34	−12.09	−2.18	4.18	−17.93	−6.69	−10.49	−1.07	−4.24	-0.69
**38**	−2.24	−1.51	−14.39	−10.47	−3.64	−11.66	−8.32	−5.08	−8.20	−11.91	−12.40	−4.53	−4.61	−5.64	−9.53	−11.34	−2.65	−3.94	−7.51
**39**	−3.53	−2.64	−19.41	−8.51	−2.35	−17.95	−11.74	−1.44	−9.49	−17.55	−10.16	−1.61	8.11	−16.42	−13.80	−6.79	−3.21	−8.19	2.66
**40**	−2.77	-0.98	−22.08	−12.72	−3.36	−7.88	4.48	−1.86	−6.34	−12.98	−12.08	−4.22	−1.32	−10.22	−5.81	−12.47	−2.26	−6.27	−7.50
**Quiz**	−4.48	−1.55	−18.63	−8.59	−7.63	−19.56	−11.13	−1.64	−10.93	−15.17	−9.37	−4.92	2.19	−9.99	−12.34	−10.03	−13.10	−7.47	−6.01
**Sunit**	−2.11	−1.65	−2.84	−10.80	−8.63	−0.98	-	−3.42	−9.61	−19.49	−12.80	-	-	−4.01	−10.82	−16.27	−19.13	−5.61	−3.63

**Table 3:** Statistical parameters evaluated in the analysis.

Parameters	Accept Values	Obtained Values
R^2^	>0.8	0.80
RMSEc	-	0.29
q^2^	>0.5	0.60
RMSEcv	-	0.46
LV	-	5
R^2^_rand_	< r^2^	0.22
RMSE _y-rand_	-	0.60
R^2^_pred_	>0.8	0.80
RMSE_pred_	-	0.31
r^2^_m(test)_	>0.5	0.68
R^2^p	>0.5	0.61

R^2^: Coefficient of determination; RMSEc: root mean square error in calibration; q^2^: Leave-one-out cross-validation correlation coefficient; RMSEcv: root mean square error in validation; LV: latent variable; R^2^_pred:_ Correlation coefficient of external validation; r^2^_m(test)_: equation 3; R^2^rand: Y-randomization; RMSE _y-rand_: root mean square error in Y-randomization; R^2^p: equation 1.

**Table 4:** Chemical structures, predicted pIC_50_ values, MolDock Scores (kcal mol^−1^), pose-protein interaction (kcal mol^−1^) and hydrogen bond interactions (kcal mol^−1^) for the most promising derivatives against FLT3.

Compd	Chemical Structure	pIC_50_ pred	MolDock Score	Inter Energy	H Bond Energy
**IAF70**	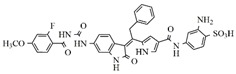	10.06	−236.96	−249.26	−9.37
**IAF72**	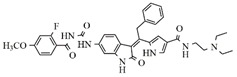	10.25	−238.17	−251.73	−6.74
**IAF75**	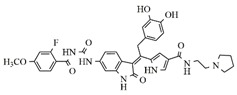	10.24	−257.27	−254.38	−13.95
**IAF80**	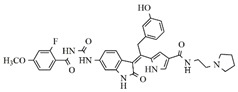	10.22	−255.22	−252.98	−11.75
**IAF84**	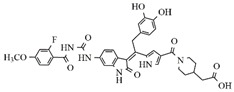	10.13	−261.42	−258.18	−11.16
**IAF88**	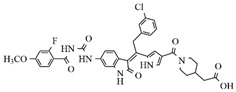	10.12	−254.29	−250.82	−8.30

**Table 5:** Chemical structure, predicted pIC_50_, MolDock Scores (kcal mol^−1^) of ligand-protein interactions (kcal mol^−1^) and hydrogen bond interactions (kcal mol^−1^) for a promising dual Aurora B/FLT3 inhibitor.

Compd	Chemical Structure	Enzyme	pIC_50_ pred	MolDock Score	Inter Energy	H Bond Energy
**IAF79**	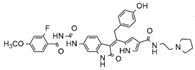	**Aurora B**	11.39 b	−236.71 b	−240.59 b	−15.61 b
		**FLT3**	9.83	−248.06	−246.77	−8.35

b [10].

## Supplementary Materials

The following are available online; Table S1: MolDock Score, Rerank Score, Pose–Protein Interaction, and Hydrogen Bond Energy values from each selected pose for compounds **1**–**40**, Quizartinib, and Sunitinib; Table S2: Measured and predicted pIC_50_ and residual values for the training compounds.
