# Supplementary material for: Theoretical Studies Aimed at Finding FLT3 Inhibitors and a Promising Compound and Molecular Pattern with Dual Aurora B/FLT3 Activity
Source: Molecules. 2020 Apr 9;25(7):1726. doi: 10.3390/molecules25071726 (PMC7181172; doi:10.3390/molecules25071726)
Supplement: Supplementary file 1 [file molecules-25-01726-s001.pdf]

Theoretical Studies Aimed at Finding FLT3 Inhibitors and a Promising Compound and Molecular Pattern with Dual Aurora B/FLT3 Activity

Ítalo Antônio Fernandes [1], Déborah Braga Resende [2], Teodorico Castro Ramalho [1,3], Kamil Kuca [3,*] and Elaine Fontes Ferreira da Cunha [1,*]

[1] Department of Chemistry, Federal University of Lavras, P.O. Box 3037, ZIP code 37200-000, Lavras-MG, Brasil; italofernad@hotmail.com (I.A.F.); teo@ufla.br (T.C.R.)

[2] Department of Veterinary Medicine, Federal University of Lavras, P.O. Box 3037, ZIP code 37200-000, Lavras-MG, Brasil; deborahbrr@gmail.com

[3] Faculty of Science, Department of Chemistry, University of Hradec Kralove, Rokitanskeho 62, 500 03, Hradec Kralove, Czech republic

* Correspondence: kamil.kuca@uhk.cz (K.K.); elaine_cunha@ufla.br (E.F.F.d.C.)

Table 1. MolDock Score, Rerank Score, Pose-Protein Interaction and hydrogen bond energy values from each selected pose for **1**-**40**, Quizartinib, and Sunitinib compounds.

Compound	MolDock Score	Rerank Score	Pose-Protein Interaction	H Bond
**1**	-233.252	-182.465	-227.406	-7.274
**2**	-195.050	-166.924	-210.154	-5.678
**3**	-197.543	-162.841	-214.397	-6.742
**4**	-191.449	-136.830	-193.104	-10.123
**5**	-219.849	-184.435	-230.886	-6.491
**6**	-212.152	-175.088	-213.269	-9.546
**7**	-193.816	-159.317	-208.211	-4.882
**8**	-213.570	-171.751	-210.209	-8.513
**9**	-183.240	-158.294	-198.502	-4.705
**10**	-189.425	-119.396	-201.969	-4.879
**11**	-186.691	-125.637	-190.609	-10.482
**12**	-209.733	-174.497	-222.323	-4.986
**13**	-186.233	-134.548	-189.394	-5.775
**14**	-207.046	-164.561	-204.653	-9.026
**15**	-189.760	-135.853	-197.143	-7.793
**16**	-182.291	-133.341	-186.034	-3.354
**17**	-172.109	-139.140	-184.718	-7.110
**18**	-199.836	-166.128	-200.421	-5.895
**19**	-188.491	-154.188	-194.903	-9.045
**20**	-190.338	-164.517	-216.916	-4.791
**21**	-190.770	-116.834	-190.710	-8.307
**22**	-176.999	-143.114	-184.734	-3.865
**23**	-201.528	-163.944	-201.077	-5.251
**24**	-186.160	-164.744	-206.209	-2.874
**25**	-169.537	-145.653	-185.638	-2.471
**26**	-163.475	-141.236	-183.672	-4.298
**27**	-187.748	-149.773	-192.222	-10.722
**28**	-172.635	-131.196	-187.467	-6.771
**29**	-160.725	-135.099	-173.663	-3.303
**30**	-183.749	-134.630	-186.908	-7.718
**31**	-172.892	-132.504	-185.770	-6.962
**32**	-197.878	-161.967	-198.980	-3.458
**33**	-164.314	-129.142	-175.389	-4.418
**34**	-189.736	-137.183	-185.379	-9.090
**35**	-156.672	-130.662	-165.637	-7.204
**36**	-185.941	-146.981	-184.351	-7.105
**37**	-165.834	-102.511	-173.414	-6.100
**38**	-192.181	-161.437	-202.810	-2.398

**39**	-158.329	-123.751	-176.726	-4.152
**40**	-167.181	-136.765	-170.554	-3.526
**Quizartinib**	-204.591	-169.283	-212.101	-1.303
**Sunitinib**	-160.944	-127.131	-167.380	-1.398

Table S2. Experimental and predicted pIC_{50}, and residual values for the training and test set.

Compound	Experimental	Predicted	Residue
**1**	9.30	8.50	0.80
**2**	8.89	8.78	0.11
**3**	8.85	8.28	0.57
**4**	8.80	8.45	0.35
**5**	8.62	8.58	0.04
**6**	8.57	8.83	-0.26
**7**	8.57	8.43	0.14
**8**	8.57	8.55	0.01
**9**	8.54	8.21	0.33
**10**	8.54	8.47	0.07
**11**	8.46	8.64	-0.18
**12**	8.36	8.22	0.14
**13**	8.32	8.20	0.12
**14**	8.21	8.17	0.04
**15**	8.14	8.62	-0.48
**16**	8.09	8.38	-0.29
**17**	8.07	7.92	0.15
**18**	8.00	8.21	-0.21
**19**	8.00	8.11	-0.11
**20**	7.99	7.87	0.12
**21**	7.94	7.54	0.39
**22**	7.81	7.84	-0.03
**23**	7.79	7.60	0.19
**24**	7.61	7.75	-0.14
**25**	7.60	7.40	0.20
**26**	7.56	7.54	0.02
**27**	7.50	7.61	-0.11
**28**	7.46	7.72	-0.26
**29**	7.43	7.67	-0.25
**30**	7.42	7.12	0.30
**31**	7.42	7.54	-0.12
**32**	7.40	6.81	0.59
**33**	7.32	7.70	-0.38
**34**	7.27	7.36	-0.10
**35**	7.19	7.18	0.01
**36**	7.13	7.01	0.12
**37**	7.06	7.21	-0.15
**38**	6.94	6.97	-0.03
**39**	6.83	7.14	-0.31
**40**	6.65	6.66	-0.01
